# Incidence of Snake Bites in Kashan, Iran During an Eight Year Period (2004-2011)

**DOI:** 10.5812/atr.6445

**Published:** 2012-08-21

**Authors:** Rouhullah Dehghani, DavarKhah Rabani, Morteza Panjeh Shahi, Mehrdad Jazayeri, Mohammd Sabahi Bidgoli

**Affiliations:** 1 Nursing Trauma Research Center, School of Health, Kashan University of Medical Sciences, Kashan, IR Iran; 2 Deputy of Health, Kashan University of Medical Sciences, Kashan, IR Iran; 3 Department of Public Health, School of Health, Kashan University of Medical Sciences, Kashan, IR Iran

**Keywords:** Snakes, Bites and Stings, Venoms

## Abstract

**Background:**

Snake bites are one of the significant health problems in the tropical and subtropical regions. Snake bite is a common medical emergency in Iran, and the epidemiological features and management of such cases vary from region to region.

**Objectives:**

This present research study was conducted to obtain new information about the epidemiology of snake bites in the region of Kashan, located in the central part of Iran.

**Patients and Methods:**

This research was a descriptive retrospective study. Data from 2004 to 2011 of snakebite cases were collected from case reports. Information included; age and sex of the victim, district, month of incident, mortality, and time of bite.

**Results:**

The results of this study showed that the majority of snake bite patients were male (96%). The age distribution of patients indicated that the greatest rate of snake bites occurred among the 15-24 year old group. Data collected in this study revealed that the highest incidence of snake bite cases took place in summer (60%) and the lowest number occurred in winter, with no snake bite cases being recorded. The peak number of snakebite cases was seen during June-September.

**Conclusions:**

It was concluded that snake bite cases in Kashan are similar to other areas in Iran from an epidemiological point of view, including; age distribution rates, gender and site of the bites. The existence of Macrov ipera lebetina, a dangerous venomous snake, can cause a range of clinical effects among residents in central parts of Iran, such as Kashan area.

## 1. Background

Venomous snakes of medical importance have a pair of enlarged, hollow teeth, called fangs, located in the front of their upper jaw. These fangs contain a venom channel (like a hypodermic needle) or groove, along which venom can be introduced deep into the tissues of their prey. If a human is bitten, venom is injected either subcutaneously or intramuscularly. Venomous animal bites are one of the significant health problem in rural populations in many parts of the world (1, 2). The consequences of snake bites, such as pain and infection can be localized or systemic, they can also induce; shock, acute kidney injury, coagulation disorders of the vascular system, rhabdomyolysis and cardiac muscle damage (3, 5). More than 3500 species of snakes have been found around the world, less than 10% of which are venomous (1, 6, 7, 8). On average, venomous snake bite incidents occur at a rate of 2.1 to 5.5 million per year, which lead to 125000 deaths and tens of thousands of chronic disabilities, mostly in Southeast Asia (9, 11). In Asia alone, it has been estimated that four million snake bites occur each year, with 50% of these envenomation attacks resulting in 100000 deaths annually (12). Snake bites occur all over Pakistan, and there are 72 species of snakes which can be found in different parts of the country (13). India has been reported to have a high annual rate of snakebites, which can reach up to 200000, and these result in between 35000 to 50000 deaths per year (3). In Nepal, an estimated 20000 snake bites occur annually and less than 200 deaths have been reported, predominantly in hospitals in eastern Terai (14, 15).In Vietnam from 1992 to 1998, an estimated 300000 snake bites were recorded per year, resulting in a death rate of 22%, which was predominantly seen among manual workers. Most of the victims were bitten by Malayan pit vipers (Calloselasma rhodostoma) (15). Papua New Guinea is the country which has one of the highest number of snake species in the world (16). In Iran, 69 species of snakes have been identified, of which 36 species are non-venomous, 25 species are venomous and 8 species are semi-venomous (17, 19). Snakebite is a serious public health problem in different parts of Iran, especially in rural areas. The recorded number of snakebites from 2001 to 2009, were approximately 5000 to 7000 per year, of which, approximately 7 deaths were reported each year in this country. Snake envenomation patterns, depending on the species, can vary amongthe four different families common in Iran, namely; Colubridae, Elapidae, Viperidae and Hydrophidae, which can cause a range of symptoms, mild envenomation, neurotoxicity, vasculotoxicity and myotoxicity (20). The severity of envenomation is divided into three levels: mild, moderate and severe. In mild envenomation symptoms include; swelling, pain, and tenderness. A moderate envenomation includes local effects such as; swelling, pain, tenderness, and systemic effects such as; nausea, vomiting, tremor, mild hypotension with evidence of coagulopathy, but no clinical bleeding. In severe envenomation local complications develop, including all organs with systemic effects such as: shock, severe bradycardia, tachypnea, or respiratory failure and coagulation disorders characterized by bleeding, and other manifestations (14, 21). The mainstay in the treatment of envenomation is antivenin, and this may be monovalent for specific species, or polyvalent, which is effective against different species. Using antivenin is only recommended for severe envenomation, since it can cause hypersensitivity reactions and these may become life-threatening (22). In some references antivenin is only recommended for moderate to severe envenomation. In the treatment of mild cases 2 to 5 vials of antivenins are normally given, in moderate cases 5 to 10 vials, and in severe intoxications 10 to 20 vials of antivenin may be needed, although in extreme cases of envenomation 45 vials may be required .In Iran, three types of antivenin, including mono, tetra and polyvalent, are produced by the Razi Vaccine and Serum Research Institute, which are used in the treatment of snake bite. The polyvalent product can neutralize the venom of six different venomous snakes (18). Due to the importance of snake bites and the small amount of epidemiological data about this public health problem, the present research study was conducted to obtain new information about snake bites in these regions. This would enable local authorities to plan strategies to reduce and eliminate snake bites among the residents of these regions.

## 2. Objectives

The purpose of this project was to conduct a retrospective study to describe the incidence and geographic location of snakebite injuries in Kashan region, and to assess the magnitude and distribution of the problem in order to optimize prevention and treatment.

## 3. Patients and Methods

This research was a descriptive retrospective study. The data of the present research came from the files of outpatient or hospitalized persons referred to the health centers and hospitals of Kashan city during an eight year period, March 22nd 2004 to March 21st 2011. In the current study, data from snake bites was studied from an epidemiology view point including; gender and age of the snake bite victim, patient background, antivenom treatment, month of snake bite, part of body bitten, and geographical location (rural/urban). The information was gathered and recorded in questionnaires. The results are presented in Tables. The frequencies of epidemiological parameters were converted to percentage ranks. During this study, many of the located viper snakes and some non-venomous snakes were collected alive, and their mouth, fangs and teeth were studied in the laboratory, to enable correct identification of the various non-venomous and venomous snakes.

## 4. Results

In total, 50 files belonging to patients who presented to health centers and hospitals in Kashan city from March 2004 to March 2011 were monitored. The incidence of snake bite in Kashan is estimated to be 2.5 persons per 100000 annually. A total of 68% of the snake bite victims were from rural areas of Kashan and the rest (32%) were from urban areas of Kashan (Table 1). The incidence of snake bite deaths in Kashan is calculated to be 2%, and one person died during the eight year period. The sex distribution results of this study showed that 96% of the snake bite patients were male and the remainder were female (4%). The age distribution of snake bite victims is presented in Table 2, which shows that the greatest rate of snake bites occurred among the 15-24 year old group, however, the lowest rate of snake bites were reported as 6% among the 10-14 and 45-54 year old patients. Snake bites were not occrued in the 0-4 and 5-9 year old age groups (Table 2). Data collected in this study revealed that the highest incidence of snake bites took place in summer (60%) and the lowest was in autumn (8%), no snake bites were recorded in winter in Kashan region. Snake bite cases reached a peak in August, but no cases were recorded from November to March (Table 3). Patients hands were the part of the body which were targeted by snake bites (60%) more than other parts, legs received 36% and the head and trunk 4% of the bites (Table 4). Out of 50 snake bite cases, 10% recovered without using antivenin serum and 88% made a recovery with an infusion of antivenin serum, in 39 cases this was administered intravenously and 6 cases intramuscular. One case (2%) of death was reported in the present study. All 10 venomous and semi-venomous snakes collected during our study from urban and rural regions of Kashan were identified as venomous snakes. These included; Macro vipera lebetina (n = 5), Pseudocerastes persicus (n = 2), Pseudocerastes persicus fieldi (n = 1), and Echis carinatus (n = 1), which belong to the Viperidae family and Malpolon monspessulanus (n = 1), which is a semi-venomous snake belonging to the Clubridae family.

**Table 1:** Annual Frequency of Snake Bite Incidence, Sex Distribution and Outcome in Cases (2004-2011)

	Snake Bite Cases, No. %	Gender		Geographical Region		Outcome	
		Male, No. (%)	**Female, No. (%)**	Rural, No. (%)	Urban, No. (%)	Recovery, No. (%)	Death, No. (%)
2004	6 (12)	6 (12)	-	2 (4)	4 (8)	6 (12)	-
2005	4 (8)	4 (8)	-	2 (4)	2 (4)	4 (8)	-
2006	4 (8)	3 (6)	1(2)	3 (6)	1 (2)	4 (8)	-
2007	9 (18)	9 (18)	-	6 (12)	3 (6)	9 (18)	-
2008	7 (14)	7 (14)	-	4 (8)	3 (6)	7 (14)	-
2009	6 (12)	6 (12)	-	6 (12)	-	6 (12)	-
2010	9 (18)	9 (18)	-	6 (12)	3 (6)	9 (18)	-
2011	5 (10)	4 (8)	1 (2)	5 (10)	-	4 (8)	1 (2)
Total	50 (100)	48 (96)	2 (4)	34 (68)	16 (32)	49 (98)	1 (2)

**Table 2:** Age Group Distribution of Snake Bite Cases Based on Gender in Kashan Hospitals and Health Centers (2004-2011)

	Male, No. (%)	Female, No. (%)	Total, No. (%)
0 - 4	-	-	-
5 - 9	-	-	-
10 - 14	2 (4)	1 (2)	3 (6)
15 - 24	13 (26)	-	13 (26)
25 - 34	8 (16)	-	8 (16)
35-44	12 (24)	-	12 (24)
45-54	3 (6)	-	3 (6)
55 - 64	7 (14)	-	7 (14)
65 ≥	3 (6)	1 (2)	4 (8)
Total	48(96)	2 (4)	50 (100)

**Table 3:** Percentage of Snake Bite Cases Based on Months and Geographical Regions of Kashan Hospitals and Health Centers (2004-2011)

	Total, No. (%)
March	-
April	2 (4)
May	6 (12)
June	8 (16)
July	8 (16)
August	15 (30)
September	7 (14)
October	4 (8)
November	-
December	-
January	-
February	-
Total	50 (100)

**Table 4:** Annual Frequency of Snake Bite Incidence, Site of Bite, Mode of Antivenin Injection and Number of Vials of Antivenin in Cases (2004-2011)

	Site of Bite			Mode of Injection			No. of Vials of Antivenin
	Hands	Legs	Head and Trunk	Venous	Muscular	No Injection	
2004	2	4	-	3	3	-	6
2005	3	-	-	4	-	-	4
2006	3	1	-	1	2	1	4
2007	5	3	-	5	1	3	6
2008	6	1	-	7	-	-	5
2009	5	1	2	5	-	1	5
2010	4	5	-	9	-	-	9
2011	2	3	-	5	-	-	14
Total, No. (%)	30 (60)	18 (36)	2 (4)	39 (78)	6 (12)	5 (10)	53

## 5. Discussion

The results of this study indicated that there were 50 cases of snake bites in Kashan during 2004-2011 years. The incidence of snake bite was 2.5 cases per 100000 in this study, and this rate is lower than the average number of snake bites in Iran which is 6.9 cases per 100000. The rate of snake bite cases had been expected to be greater than the observed data found in Kashan area, as the regional climatic conditions produce a warm, arid climate. The high level of awareness by the local people and health authorities concerning health problems such as snake bites has been suggested as the reason for the lower rate of snake bites, combined with well functioning pest control. Results of this study showed that most of the snake bite patients were male (96%) and only a few were female (4%). This means that males are at greater risk of snake bites than females in Kashan. The male to female ratio was 24:1. This is in accordance with results found by a number of different researchers in India (23, 25, 26), however, these ratios were in contrast with the results from other authors which are rather higher. Studies from other countries also indicate a preponderance of male victims, the male: female ratio was reported as 1.9:1 in Thailand and 1.3:1 in Pakistan (27). Nhachi et al. and Paul et al., on the other hand, reported that females were at increased risk of snake bite compared to males. The predominance of male victims suggests an increased risk from outdoor activities. The sex ratio seems to be almost uniform in all the earlier reports, with males being affected twice or three times more often than females (28, 29). In a study by Tanen et al. the majority of snake bite victims were adult males (30), it has been proposed that the epidemiology of snake bites is related to gender and job type. Another important epidemiologic factor which was found in the current research is the location where the snake bite occurred, in a rural or urban area. It has been reported that 68% of snake bite cases occur in rural areas of Kashan. This agrees with the results of Sharma et al. in India. It was reported, that the majority of the snakebites (82%) take place among the rural population (16) who are bitten in agricultural fields while working and also while sleeping outdoors (31). The greatest rate of snake bites occurred among people aged 15-24 years old, and the lowest rate was reported as 6% among the 10-14 and 45-54 year old patients in this study. Snake bites did not occur in age groups 0-4 and 5-9 years old, and this agrees with the findings of previous researchers (24, 27). However pooled age data of 15-24, 25-34 and 35-44 year old as one group of 15-44 year olds included 66 snake bitten patients because this age group included the highest working age group. Therefore this age group had greater risk factors for snake bites than the other groups. Data collected in the current study revealed that the highest incidence of snake bite cases took place in summer (60%). This is in accordance with the studies of Srihari et al. and Brunda and Sashidhar in India (24, 32). Brunda and Sashidhar reported that 49.7% - 93.4% of snake bite cases occur in summer. These differences were presumably due to variations in geographical, climatic and species distributions. In the present study, the highest number of bites was recorded between June and September and this is similar to that recorded earlier by Pondichery (32). The possible reason for the majority of snakebites in the hot season may be attributed to the greater activity of snakes at this time, as they are cold blooded animals. Consequently, the human population becomes accidental victims to snakebites. Moreover, the situation is aggravated by the propensity of rodents, lizards and other potential prey to be located near human habitats and places of work, thus increasing the risk of snake-bite (24). The results of this study revealed that limbs which are moving parts, are at higher risk of snakebites compared with the head and trunk. This suggests that people working in affected areas, need to wear suitable protective clothing, as this is an important issue in the reduction of snakebite cases. This result is similar to other studies, in the predominance of envenomations in the upper extremities (30, 33). It is concluded that snake bites in Kashan is similar to other areas from an epidemiological viewpoint of including; age distribution, gender and site of bites. The lower rate of snake bite cases in Kashan, than in the south of Iran could be due to the different species of snakes and the types of buildings in urban and rural area. Another reason related to the lower number of injuries and mortality may be related to the job distribution in Kashan, which was not studied in this research. Therefore, it is suggested that the type of work that people in Kashan undertake should be considered in future research. To continue a reduction in the rates of snake bite among the people of Kashan, further education needs to be considered; with special emphasis put on emergency medical staff for identification of venomous and non venomous snakes is an essential first step. Developing a standard protocol for native snake species could make a significant improvement in the treatment of envenomation cases; this would decrease the seriousness of the patient’s resulting injury and also reduce operating costs.
